# Behind the scene of the prevalence of anaemia: an extended way of reporting

**DOI:** 10.1017/S1368980023000393

**Published:** 2023-06

**Authors:** Sabuktagin Rahman, Nazma Shaheen

**Affiliations:** 1 Department of Nutrition and Food Engineering, Faculty of Allied Health Sciences, Daffodil International University, Birulia, Savar, Dhaka-1216, Bangladesh; 2 Griffith University, Public Health, School of Medicine and Dentistry, Gold Coast, QLD, Australia; 3 Institute of Nutrition and Food Science, University of Dhaka, Dhaka, Bangladesh

**Keywords:** Anaemia, Extended reporting, Animal source food, Thalassaemia, Groundwater Fe

## Abstract

**Objective:**

To develop the methods for an extended reporting of anaemia and to measure the status of the key contextual underlying factors of anaemia.

**Design:**

Statistical appraisal of Hb *v*. key influencers of anaemia in Bangladesh – the intake of animal source food (ASF), concentration of Fe in the drinking groundwater (GWI) and the prevalence of congenital Hb disorder (CH) are conducted. The primary data of the National Micronutrient Survey 2011–2012 and the British Geological Survey 2001 are analysed to assess the intake of ASF and the GWI concentration, respectively. The prevalence of thalassaemia from a national survey is used to appraise the CH. ASF is evaluated relative to the 97·5^th^ percentile intake and group scores are assigned. Association of the GWI and Hb is examined by the linear fit and the mspline fit and the group scores are allocated. Group score is allocated for the prevalence of thalassaemia. Inflammation-adjusted ferritin is considered to report Hb.

**Setting:**

A nationwide survey in Bangladesh.

**Participants:**

Preschool children (6–59 months), school-age children (6–14 years) and non-pregnant non-lactating women (NPNLW, 15–49 years).

**Results:**

The extended reporting to the prevalence of anaemia in Bangladeshi preschool children, school children and women is – anaemia 33 % (ASF: 2·08; GWI: 1·75; CH: 2), anaemia 19 % (ASF: 1·98; GWI: 1·56; CH: 2) and anaemia 26 % (ASF: 2·16; GWI: 1·58; CH: 2), respectively.

**Conclusion:**

The extended reporting of anaemia is a useful tool to understand the status of the key influencers of anaemia, to design the context-customised intervention and to monitor the intervention.

## Graphical abstract

**Figure:**
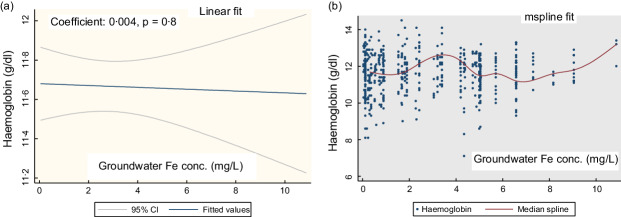

Anaemia is one of the most prevalent public health problems worldwide. A recent estimate suggests nearly 2 billion people suffer from anaemia globally^(1)^. Public health consequences of anaemia are profound, such as physical weakness, poor growth and low attention span in children; low educability and lower productivity in adulthood^(1,2)^. It causes obstetrical complications during childbirth and linked with maternal and infant mortality. Causes of anaemia are multiple – quantitative and qualitative deficiency of diet, low animal protein, low intake of micro-nutrients such as Fe, vitamin A, vitamin B_12_, Zn, folate and vitamin C^(2–4)^. Environmental- and disease-associated factors such as infection and inflammation are the other causes of anaemia, so as the congenital Hb disorders such as thalaessaemia and Hb E disorders. Malaria is a prominent cause of anaemia in many settings, particularly in Africa^(5,6)^. In Bangladesh and some other countries, Fe acquired through the groundwater source of drinking has emerged as a determinant of Fe and anaemia status in the population^(7,8)^.

Amid the heterogeneous causes of anaemia, it is estimated by one unique standard – the prevalence in population, i.e. the proportion of population with Hb concentration by age, gender and physiological status specific cut-offs to report anaemia. The prevalence estimate solely provides the data on the magnitude of the condition and is insensitive to its diverse causes. Globally, the prevalence estimate of anaemia gets the high attention, and it promptly decides the intervention. This often leads to the intervention that does not optimise to the dominant factors of anaemia in a particular setting. Nonetheless, in resource-constrained settings where prevalence of anaemia and ID is high, Fe supplementation is essentially recommended for certain groups such as young children and pregnant women. On the other hand, evidence base is growing that ID is not a dominant cause of anaemia in some settings^(7,8,9)^, since these populations are naturally replete in Fe status from the drinking groundwater sources which contains a fair amount of bioavailable Fe. In many settings, thalassaemia is prevalent which is associated with low prevalence of ID^(10,11)^. In these settings, the blanket Fe supplementation might lead to excess Fe and the side effects^(12,13)^. Therefore, it is essential to understand the context-specific determinants underlying anaemia. Reporting of the prevalence of anaemia supplemented with some key data predominantly associated in a given context may enable an objective and judicious way to control anaemia. Therefore, in the present study, we attempted to characterise the reporting of anaemia beyond the prevalence estimate, by supplementing with some select data which will shed lights into the status of the key correlates of anaemia in a given setting. Through inclusion of the key contextual data within a parenthesis next to the prevalence estimate, the extended reporting would provide the valuable information to the policymakers, academicians and nutrition programme managers for precise planning and monitoring of the programme actions.

## Setting

The study is conducted in Bangladesh a South Asian low-mid-income country with a medium Human Development Index. Prevalence of anaemia in under-five children is 30–33 % according to different national micronutrient status surveys^(14,15)^. The prevalence of stunting in the under-five children is 28 %^(16)^. Traditional dietary practice is predominantly cereal based which provides approximately 70 % of the calories,^(17)^ and the bio-availability of Fe is sub-optimum^(18)^. Intake of animal source food (ASF) has been increasing over the last decade but still it is remaining below the optimum intake. Thalassaemia is prevalent as the country lies within the thalassaemia belt regions. Thalassaemia is a congenital disorder of Hb synthesis, characterised by anaemia which often remains non-responsive to Fe intervention^(19)^. Groundwater is the principal source of drinking water of Bangladeshi population, and it contains a fair-to-high amount of high absorbable Fe. It has been shown that the groundwater Fe *v*. Fe and Hb status is positively associated in different population groups^(7,8,9,14,20,21,22)^. Magnitude of the infection burden, such as the acute respiratory infection and the concentration of Hb, are associated^(23)^. The burden of common infection is present but acceptable given that Bangladesh is a densely populated subtropical country. At the national level, the CRP > 5 mg/l is 4·9–9·5 % across the population groups signifies that the overall infection burden is low^(14,24)^ compared with settings which have a high infection burden and/or malaria is endemic.

On this background setting, the methods for the extended reporting of anaemia using the published data of the national micronutrient survey 2011–2012 are developed^(14)^. The national prevalence of anaemia in Bangladesh – 33 % in preschool-age children (PSC) (6–59 months), 19 % in school-age children (SAC) (6–14 years) and 26 % in non-pregnant non-lactating women (NPNLW) (15–49 years)^(8,14)^. The prevalence is supplemented with the extended reporting on the status of the prevailing correlates of the Hb status in population.

## Methods

Taking into consideration the prominent determinants of Hb status, the three factors in Bangladesh context need assessment for possible inclusion in the extended reporting of anaemia. These are diet, groundwater Fe and congenital Hb disorders.

### The basis for the dietary factor

Hb has two parts – he haeme moiety containing four atoms of Fe and the protein component globin containing two polypeptide chains^(25)^. ASF is the source of first-class protein,^(26)^ which is important for the synthesis of globin chain of Hb. ASF contains haeme-Fe, retinol, Zn and vitamin B_12_, which have documented haemopoetic potential^(26)^. Haeme Fe has a high bioavailability among the sources of dietary Fe^(18)^. Fe is needed for the synthesis of haeme molecule of Hb. Retinol has a higher bio-conversion among different genres of vitamin As^(26)^. A positive association of serum vitamin A status and Hb is documented in Bangladesh^(8)^. Vitamin B_12_ is essential for the synthesis of the globin component of Hb and is found exclusively in ASF^(26)^. These essential nutrients of haemopoietic potential contained in the ASF make the latter a suitable dietary component for assessment for inclusion in the extended reporting of anaemia.

ASF-derived micro-nutrients are not considered, as the Kendall’s tau b correlation reveals that the coefficients of Hb *v*. animal source Fe, Hb *v*. animal source vitamin A and Hb *v*. animal source Zn are very small and statistically non-significant in PSC and NPNLW (see online Supplementar Table 1), precluding them from selection for the extended reporting. Second, to avoid the difficulties in the estimation of micro-nutrient intake which might be cumbersome in the settings with limited technical resources, including the paucity of local food-based food composition tables.

### The rationale for the groundwater iron and congenital Hb disorders

Groundwater Fe is explored for the extended reporting as the water source is the predominant type of drinking water in Bangladesh^(27)^. Furthermore, several studies have shown the favourable association of groundwater Fe and Hb and/or anaemia^(7,8,9,14,20,22)^.

Globally, thalassaemia accounts for over 17 % of the 330 000 infants born annually with congenital Hb disorders. It accounts for nearly 3·4 % of deaths in children less than 5 years of age^(28)^. The prevalence is higher in the particular regions – the Mediterranean, North Africa, the Mid-East and the North-East of the Indian Sub-Continent and the Indo-China peninsula^(29)^. Unfavourable association of the congenital Hb disorders, e.g. thalassaemia, Hb E conditions and anaemia/Hb concentration is documented in Bangladesh^(30)^. Merrill *et al.* have shown that thalassaemia is independently associated with anaemia in Bangladeshi women of reproductive age after controlling for the confounders (adjusted OR: 2·48, 95 % CI (1·24, 4·94), *P* < 0·05)^(30)^.

The common infectious diseases (e.g. diarrhoea and common cold) are not considered as these are within the acceptable level consistent with a developing country context, e.g. prevalence of diarrhoea ∼5 %^(31)^. Moreover, the magnitude of the infection burden is not large^(14)^ as in many other settings^(32)^. Malaria is not considered as it is non-endemic in Bangladesh.

The consumption of ASF is analysed from Bangladesh national micronutrient status survey 2011–2012 (NMS 2011–2012). Groundwater Fe data of a nationally representative hydrology survey^(27)^ are extrapolated to the NMS 2011–2012 data to analyse the water Fe component. The prevalence data of congenital Hb disorders of a nationally representative survey are used to develop the thalassaemia component^(33)^.

### Specific approaches and statistical analysis

#### Animal source food and Hb

The non-parametric correlational analysis – Kendall’s tau b is used to assess the association of the intake of ASF and Hb concentrations in the populations.

Mean daily intake of ASF is estimated by a validated semi-quantitative FFQ used in the national micronutrient survey 2011–2012. Commonly consumed ASF in Bangladesh are considered, e.g. small fish, large fish, chicken, beef, mutton, eggs, milk, butter and cheese. The data on the absolute number of times that the particular food is consumed over the preceding week and the average amount each time the food is consumed are collected. The measured amount of weekly food consumption (i.e. cooked food) is converted to daily average intake^(34)^. The 97·5^th^ percentile of the daily intake is estimated. The 97·5^th^ percentile of the intake of ASF does not refer to the RDA, as there is no RDA for ASF. In Bangladesh, 70 % of calories are consumed from the cereal-based foods^(17)^. The content of protein in Bangladeshi ASF (fish, meat and eggs) ranges from ∼13 to 22 g/100 g of raw weight^(35)^. The content of protein in the cereals and legumes, two plant-foods with high consumption, range ∼12–25 g/100 g of raw weight. Considering the revised dietary reference of protein intakes – children 1·3–0·8 g/kg body weight and adults 0·8 g/kg body weight^(36)^ and the average body weight of 12·5 kg in 2–5 year-old children^(9)^ and 49 kg in non-pregnant women^(14)^, this translates a requirement of protein of 10–16·25 g/d and 39·2 g/d, respectively. Hence, the calculated 97·5^th^ percentile intake (395–449 g/d) of the measured (i.e. cooked) ASF states an upper ceiling of sufficient intake against which the actual intake will be measured in the extended reporting of anaemia.

Calculation of the pre-defined sub-groups of ASF intake is done sorted by ranges of proportions relative to the 97·5^th^ percentile. The range of the proportion, i.e. 0–100 % relative to the 97·5^th^ percentile intake is sub-grouped, and each group is allocated with a numerical score; <25 % = 1; 25–37·5 % = 2; 37·5–50 % = 3; 50–62·5 % = 4; 62·5–75 % = 5; 75–87·5 % = 6 and >87·5 % = 7.

Kruskall–Wallis Equality of Population Rank Test is performed to assess the relative magnitude of the sub-groups. For the intake of ASF, the fweight command in STATA (STATA 13) is used to calculate the weighted average of the group scores (i.e. - score). A higher weighted average score (on a scale 1–7) is indicative of a higher intake of ASF relative to the 97·5^th^ percentile.

#### Groundwater iron and Hb

The primary data set of the NMS 2011–2012 lacks the data of groundwater Fe concentration. The groundwater Fe data of Bangladesh are curated from the British Geological Survey 2001. The mean concentration of groundwater Fe is estimated at the sub-district level (results not shown), and the mean is extrapolated to the sub-district level of the NMS 2011–2012 data. Therefore, the data of the drinking water Fe concentration of the participants are not the direct data of their drinking source, but an approximated average of the sub-district. Sub-districts are sub-grouped into three categories, classified by the groundwater Fe concentration – <2 mg/l, 2–10 mg/l and ≥10 mg/l^(27)^. Spearman rank correlation coefficient (rho) of groundwater Fe concentration and Hb is estimated to assess the association. The linear fit is constructed for the groundwater Fe concentration on the x-axis and Hb concentration on the y-axis. Furthermore, mspline line graphs are computed. The mspline is the interpolated cross-medians of the groundwater Fe and Hb concentrations grouped by ten bands (i.e. regions) on the x-axis. This provides a specific zone-wise relationship of the variables which is often not captured in the linear-fit model. The mspilne line graphs categorised by the water Fe concentrations is utilised to allocate numerical scores – 1, 2 and 3 over the sub-groups – <2 mg/l, 2–10 mg/l and >10 mg/l. Weighted mean score is calculated for the association of groundwater Fe concentration and Hb on a scale 1–3. A higher weighted average score suggests favourable influence of the water Fe, thus a protective role against the low level of Hb.

Since, inflammation affects adversely the concentration of Hb^(37,38)^, ferritin which is closely associated with Hb is adjusted for inflammation by excluding the cases of Hb from analysis when C-reactive protein (CRP) is >1 mg/l^(24)^.

#### Congenital Hb disorder and Hb

In Bangladesh, various studies indicated varied prevalence of the condition — 2·9–16 %^(39)^, 17·2 %^(40)^, 13·1 %^(9)^ and 28 %^(7)^. A recent nationally representative survey reported the combined prevalence of Hb E (ETT) and thalassaemia B traits (BTT) was 11 %^(33)^. A review of the global burden of thalassaemia reported that the dominant frequencies of *β*-thalassaemia carrier and Hb-E disorders were <10 % and 10–25 % over most of the countries. A few of the countries exceeded the prevalence>25 %^(41)^. Hence, we propose the following operational grade score for the effect of the congenital Hb disorders on Hb status – prevalence<10 % = 1, prevalence 10–25 % = 2 and prevalence>25 % = 3. If the area-representative prevalence studies are conducted over multiple areas within the country, the weighted grade scores need to be calculated. A higher grade-score or the higher weighted grade-score (on the scale 1–3) is indicative of the burden of the congenital Hb disorders suggesting a low protection from the low Hb level.

Hb is measured by a portable photometer, Hemocue 301 (Hemocue AB, Angleholm, Sweden) on venous blood sample. Ferritin and CRP is measured by Sandwich ELISA at the Nutritional Biochemistry Laboratory of the International Centre of Diarrhoeal Diseases Research, Bangladesh (ICDDR,B). Ferritin is adjusted for inflammation following the method shown by Rahman *et al*.^(24)^ Groundwater Fe concentration is measured by the inductively coupled plasma atomic emission spectrophotometry (ICP-AES)^(27)^.

The primary data of the national micronutrient survey 2011–2012 is obtained through permission of the National Nutrition Services, Directorate General of Health Services, Ministry of Health, Government of Bangladesh. Ethical clearance for the survey is provided by the Institutional Review Board of ICDDR,B. Written informed consent of the parents of the children is taken prior to data collection. For the appraisal of the groundwater Fe concentration in Bangladesh, the primary data of the British Geological Survey 2001 is used upon permission of the British Geological Survey^(27)^.

## Results

### Animal source food *v*. Hb

Table 1 shows the intake of ASF and Hb are positively correlated when ferritin is unadjusted for inflammation. There is a slight modification of the association, but still remains statistically significant when ferritin is adjusted for inflammation.

**Table 1:** Association of the intake of animal source food (ASF) and Hb concentration in Bangladeshi population

Population	Hb with the ferritin unadjusted for inflammation			Hb with the ferritin adjusted for inflammation*		
	*n*	Kendall’s tau *b*	*P* value	*n*	Kendall’s tau *b*	*P* value
PSC†	569	0·15	0·0000	297	0·13	0·001
SAC‡	1323	0·06	0·0011	1066	0·05	0·02
NPNLW§	1036	0·05	0·05	497	0·035	0·06

* The exclusion of the cases of Hb from analysis when C-reactive protein (CRP) was >1 mg/l^(24)^.

† Preschool children.

‡ School-age children.

§ Non-pregnant non-lactating women.

#### Intake of animal source food at the 97·5^th^ and 100^th^ percentiles in the Bangladeshi populations

The 97·5^th^ percentiles of intake of ASF are 449·34 g/d, 426·43 g/d and 395·07 g/d in Pre-School Children, School Age Children and Non-Pregnant Non-Lactating Women, respectively (see online Supplemental Table 2).

Table 2 presents the sub-groups by proportion ranges relative to the 97·5^th^ percentile intake of ASF in PSC, SAC and NPNLW. Sub-groups with progressively higher intake of ASF are allocated with higher grade-scores on a scale from 1 to 7. Size of the sub-groups differs with statistical significance at *P* < 0·000.

**Table 2:** Assessment of the relative size of the sub-groups of the animal source food (ASF) intake in the Bangladeshi populations

ASF sub-groups	Sub-groups by proportion ranges relative to the 97·5^th^ percentile intake of ASF	Group score	*n* †	*χ* ^2^ with ties	*P* value
PSC					
ASF1*	<25 %	1	496	570·74	0·0001
ASF2	25–37·5 %	2	95		
ASF3	37·5–50 %	3	49		
ASF4	50–62·5 %	4	36		
ASF5	62·5–75 %	5	32		
ASF6	75–87·5 %	6	33		
ASF7	>87·5 %	7	35		
SAC					
ASF1*	<25 %	1	863		
ASF2	25–37·5 %	2	237		
ASF3	37·5–50 %	3	98		
ASF4	50–62·5 %	4	51	1074·07	0·0001
ASF5	62·5–75 %	5	54		
ASF6	75–87·5 %	6	44		
ASF7	>87·5 %	7	60		
NPNLW					
ASF1*	<25 %	1	767		
ASF2	25–37·5 %	2	257		
ASF3	37·5–50 %	3	127		
ASF4	50–62·5 %	4	62	1158·75	0·0001
ASF5	62·5–75 %	5	69		
ASF6	75–87·5 %	6	51		
ASF7	>87·5 %	7	67		

PSC, preschool children (2–5 years old); SAC, school-age children (6–14 years); NPNLW, non-pregnant non-lactating women (15–49 years).

* ASF1–7: Seven categories of ASF intake determined by the percentile ranges relative to the 97·5^th^ percentile intake.

† Kruskall–Wallis equality of population rank test was performed to assess the relative magnitude of the sub-groups.

Table 3 shows the weighted scores of the intake of ASF in Bangladeshi populations. The mean scores are 2·08 ± 1·72, 1·98 ± 1·65 and 2·16 ± 1·73 in PSC, SAC and NPNLW, respectively.

**Table 3:** Weighted score of intake of animal source food (ASF) in Bangladeshi populations

Population	Weighted score*,† of intake of ASF		
	*n*	Mean	sd
PSC	816	2·08	1·72
SAC	1407	1·98	1·65
NPNLW	1400	2·16	1·73

ASF, animal source food; PSC, preschool children (2–5 years old); SAC, school-age children (6–14 years); NPNLW, non-pregnant non-lactating women (15–49 years).

* On a scale of 1–7.

† fweight command in STATA was used to calculate the weighted average of the group scores.

### Groundwater iron and Hb

**Fig. 1:**
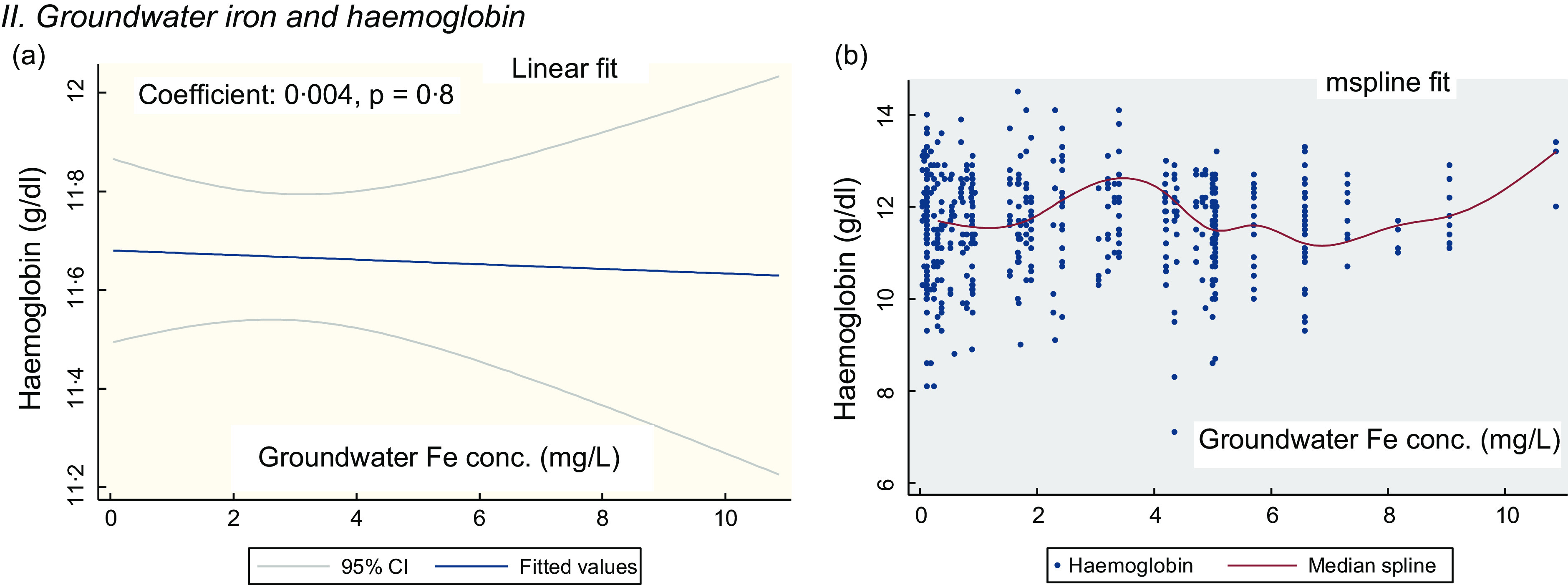
The regression association of the concentration of the groundwater iron and Hb in preschool children (PSC)

Figure 1(a) shows the linear fit for the groundwater Fe concentration and Hb in PSC. There is no linear association (coefficient: 0·004, *P* = 0·8). The mspline cross-medians (Fig. 1(b)) for the groundwater Fe concentration and Hb shows the relatively low trend of Hb up to the groundwater Fe concentration 2 mg/l. Over the water Fe concentration 2–8 mg/l, the trend of Hb is upwards and downwards. At around the water Fe concentration 10 mg/l, there is an increasing trend of Hb concentration.

**Fig. 2:**
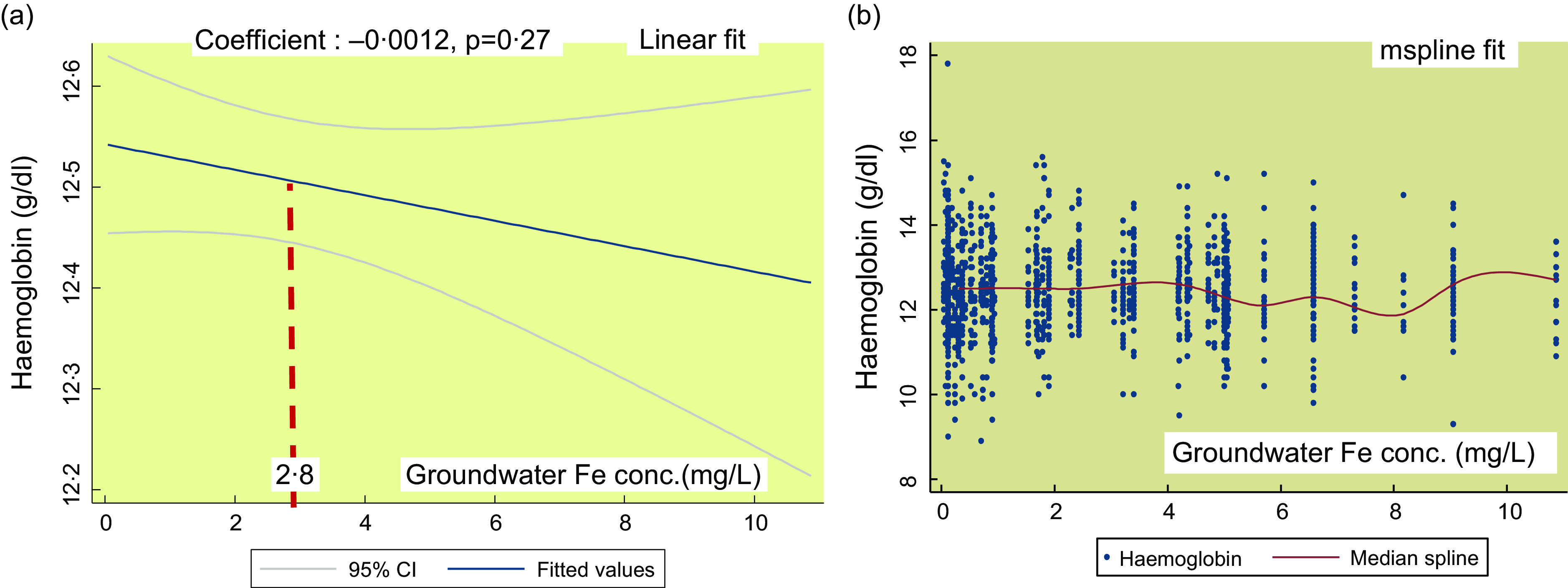
The regression association of the concentrations of the groundwater iron and Hb in school-age children (SAC)

Figure 2(a) shows the linear fit for groundwater Fe concentration on Hb in SAC. There is no significant association (coefficient: -0·0012, *P* = 0·27). The mspline, i.e. cross medians of water Fe concentration and Hb (Fig. 2(b)) show that there is a steady trend of Hb concentration up to the groundwater Fe concentration 7 mg/l. Around the 8–10 mg/l range, the Hb concentration shows a rising trend.

**Fig. 3:**
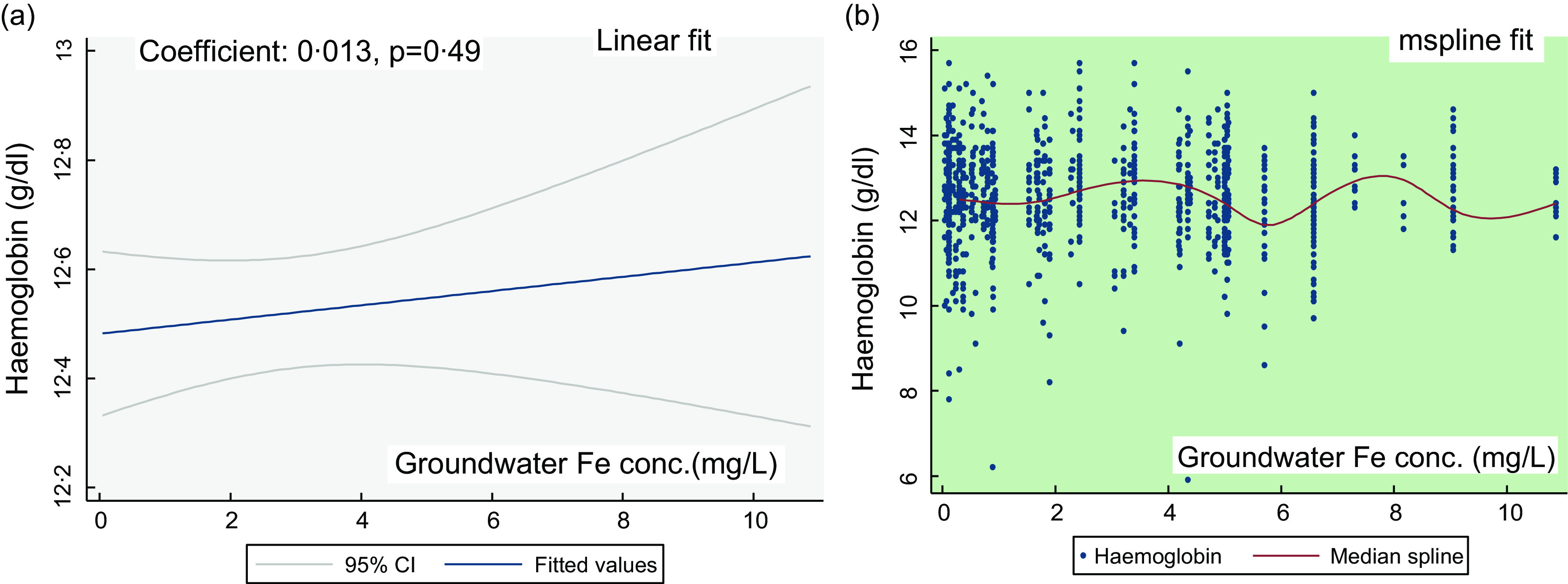
The regression association of the concentrations of groundwater iron and Hb in non-pregnant non-lactating women (NPNLW)

Figure 3 shows the linear fit for the groundwater Fe and Hb concentrations in NPNLW. There is a statistical non-significant association (coefficient: 0·013, *P* = 0·49; Fig. 3(a)). The mspline cross-medians (Fig. 3(b)) for the groundwater Fe concentration and Hb depicts a rising trend of Hb concentration over the 2–4 mg/l range followed by a drop; and a rise over the 6–8 mg/l mark. At around 10 mg/l, the trend of the concentration of Hb is observed to be rising.

Table 4 depicts the Spearman Rank correlation of the groundwater Fe and Hb concentrations, the provision of the group-scores; and the estimation of the weighted scores for the association of groundwater Fe and Hb. Spearman Rank correlation coefficient (rho) is small and statistically non-significant.

**Table 4:** Correlation of the groundwater iron and Hb concentrations and the estimation of the weighted scores

Sub-groups by iron concentration in groundwater*	Correlation GWI conc *v*. Hb		Group scores†	Weight estimation		
	Spearman rho, n1	*P* value		N2		Weighted-average score‡
PSC						
<2	−0·06, 150	0·47	1	286		
2–10	−0·10, 106	0·27	2	362	1313/749	1·75
>10	−0·36, 28	0·05	3	101		
SAC						
<2	−0·09, 535	0·04	1	722		
2–10	−0·02, 374	0·67	2	505	2134/1361	1·56
>10	−0·16, 104	0·1	3	134		
NPNLW						
<2	−0·05, 225	0·40	1	701	2125/1347	1·58
2–10	−0·003, 191	0·96	2	514		
>10	−0·03, 48	0·80	3	132		

PSC, preschool children (2–5 years old); SAC, school-age children (6–14 years); NPNLW, non-pregnant non-lactating women (15–49 years).

* The groundwater levels are sub-grouped into three categories determined by the groundwater Fe (GWI) concentration – <2 mg/l, 2–10 mg/l and ≥10 mg/l^(27)^.

† The group scores are the operational allocation of scores to the categories of the GWI concentration.

‡ Weighted average scores on the GWI concentration are derived by two steps – a. multiplying the group size by the corresponding allocated group scores followed by summation of the product [X], b. summation of the groups size [Y]. Weighted average score = X/Y^(42)^. It determines a weighted average of the group scores for the effect of GWI concentration on Hb.

The average weighted scores for the association of the groundwater Fe and Hb are 1·75, 1·56 and 1·58 in PSC, SAC and NPNLW, respectively. A higher weighted score underscores the higher degree of favourable influence of the groundwater Fe on Hb.

### Thalassaemia and Hb

Taking into consideration of the national prevalence of the carrier state of 11 %^(33)^ and the ranges of the burden of the condition (in the Methods section), the grade score 2 is allocated for congenital Hb disorders (CH) for Bangladesh.

Table 5 shows the extended reporting to the prevalence of anaemia in Bangladesh setting. In PSC, the weighted scores for the intake of ASF and groundwater Fe concentration are 2·08 and 1·75, respectively. In SAC, the respective scores are 1·98 and 1·56; while in NPNLW, the scores are 2·16 and 1·58, respectively. The score for the congenital Hb disorder is 2 in all the population groups.

**Table 5:** Extended reporting of anaemia in Bangladesh

Population	Prevalence of anaemia*	Weighted score† on the ASF§	Weighted score‡ on the groundwater iron‖	Score§ on congenital Hb disorders¶	Extended reporting of anaemia
PSC	33 %	2·08	1·75	2	Anaemia 33 % (ASF: 2·08; GWI: 1·75; CH: 2)
SAC	19 %	1·98	1·56	2	Anaemia 19 % (ASF: 1·98; GWI: 1·56; CH: 2)
NPNLW	26 %	2·16	1·58	2	Anaemia 26 % (ASF: 2·16; GWI: 1·58; CH: 2)

PSC, preschool children (2–5 years old); SAC, school-age children (6–14 years); NPNLW, non-pregnant non-lactating women (15–49 years); ASF, animal source food; GWI, groundwater Fe; CH, congenital Hb disorders.

* NMS 2011–2012.

† fweight command in STATA was used to calculate the weighted average scores on the ASF.

‡ Weighted average scores on the GWI concentration are derived by two steps – a. multiplying the group size by the corresponding allocated group scores followed by summation of the product [X], b. summation of the groups size [Y]. Weighted average score = X/Y^(42)^. It determines a weighted average of the group scores for the effect of GWI concentration on Hb.

§ On a scale 1–7.

‖ On a scale 1–3.

¶ On a scale 1–3.

## Discussion

The study attempts to provide an extended reporting of anaemia beyond the prevalence estimate. The intent is to report the relative status of the key underlying factors (favourable or unfavourable) of anaemia in Bangladeshi population. Methods to assess the status of the three key correlates that have documented association with anaemia in Bangladeshi population are developed, and the status of the factors is evaluated.

### Interpretation of the extended reporting

#### Animal source food *v*. Hb

A higher weighted score on ASF (on a scale of 1–7) is indicative of the relative protection from the low level of Hb. In PSC, the weighted score 2·08 suggests that the intake of ASF is above 37·5 % relative to the 97·5^th^ percentile. In SAC, the score of 1·98 indicates that the intake is short of 37·5 % relative to the 97·5^th^ percentile. In NPNLW, the weighted score 2·16 suggests that the intake is 40·5 % relative to the 97·5^th^ percentile. The results are revelatory of sub-optimum intake of ASF across the population groups.

#### Groundwater iron concentration *v*. Hb

The observation of predominantly high trend of Hb at around the water Fe concentration 10 mg/l, a mix up over the middle range and a flatter trend of Hb at the groundwater Fe concentration <2 mg/l (Fig. 1(b) and 3(b)) are considered for the allocation of the group scores. This is complemented by the rising and/or higher trend of the inflammation adjusted ferritin starting at around the 8–11 mg/l mark (see online Supplemental Fig. 1). A higher weighted score for groundwater Fe is suggestive of higher relative protection from the low level of Hb. In PSC, the weighted score 1·75 suggests that the children are fairly protected from the low level of Hb. In SAC and in the NPNLW, the scores (1·56 and 1·58, respectively) indicate a fair degree of protection; nonetheless, the scores are somewhat lower than in the PSC. The difference is difficult to explain. However, at the groundwater Fe (GWI) concentration ≥10 mg/l, there is a high trend of Hb concentration. To complement this, the proportion of the subjects exposed to the water Fe concentration ≥10 mg/l is slightly higher in PSC (13·5 %) than in the SAC (9·79 %) and NPNLW (9·84 %) (Table 4). This possibly accounts for higher score for the water–Fe in the PSC. The preschool age children drink much lower amount of drinking water compared with women or older children. This hints that their intake of Fe from groundwater is lesser, and thus they might have relatively a lower reserve of body Fe than in the older children or adult women. The lower reserve of body Fe possibly induces an efficient hepcidin-mediated absorption of Fe^(43,44)^. Hence, in PSC compared with other two groups, the Hb concentration can be slightly higher responsive to GWI concentration, which may confer a better protection.

The GWI influencing the Hb concentration is a structural issue in the population. Natural Fe content in groundwater is a fixed geological phenomenon, as such that the protection from the low level of Hb in the population is likely to continue over the time. The profoundness of this association is evident from the findings that the presence of any level of Fe in groundwater is associated with Hb concentrations mostly above the cutoff for anaemia in all three population groups (Fig. 1(b), 2(b) and 3(b)). However, the probable cause of the small coefficient of association of GWI and Hb concentrations (Fig. 1(a) and 3(a), Table 4) can be a fair preexisting level of Hb, so much so, that the additional exposure to the GWI yields a subtle effect on Hb. The analysis from one of our different projects suggests that in preschool children at a very low concentration of Fe in groundwater (<0·8 mg/l), Hb is maintained above the cut-off for anaemia.

#### Congenital Hb disorders and Hb concentration

A higher score for congenital Hb disorders is indicative of lesser protection from the low level of Hb. The score reported in the present study suggests an intermediate degree of influence with which the Hb level is at the risk of getting low.

Among the three factors considered, GWI and thalassaemia disorders exert the fixed structural influence on Hb concentration/anaemia. The former affects favourably and the latter affects adversely. Given the low weighted score of the ASF, the ASF is the potential area where increasing the intake has the scope to improve the score, and thus may improve the prevalence of anaemia.

Supplementation/fortification of Fe is not considered in this reporting, as the focus is on the key structural and primary influencing factors pertaining to anaemia. Nonetheless, the use of supplements/fortification may be guided by the findings of the extended report. Such as in the cases of the presence of the structural factors, i.e. GWI and congenital Hb disorders which have strong influence on anaemia; caution should be exercised implementing the supplementation/fortification programmes. In the presence of the structural factors, the supplemental/fortified Fe may induce excess load of Fe, which is associated with side effects emanated from the unhealthy composition of gut microbiome^(45,46)^. The Fe supplementation programme may be implemented in the particular areas where the baseline Fe status of population is sub-optimum, or the programme intervention may be modified, e.g. changing the dose of the iron supplements according to Fe status in population and/or the groundwater iron concentration.

In the countries/settings where malaria is endemic, the proportion estimate of the global caseload^(47)^ can be graded to derive a score for malaria. Globally, this proportion of the global caseload is highly variable. In some countries of Africa, this estimate is over 25 %, while in other countries it is around 5 %^(47)^. Hence, we suggest the following operational cut-offs for the grade scoring—case burden in the country <2 %=1, 2–5 %=2, >5 %=3. In the extended reporting format, malaria can be reported as ‘M’ in the short form.

### Guidelines for usage

- The underlying principle of selection of the factors in a particular setting is the factor that show the local evidence of association with anaemia, and these should be the structural/key influencing factors. For a given setting, if groundwater is a significant source of drinking, GWI should be measured and included in the extended reporting of anaemia. Fe concentration in water can be measured either by the portable colourimetric devices or atomic absorption spectrophotometry. Regarding the CH, in case of any anaemia assessment study or anaemia intervention programme, the prevalence of CH needs to be assessed since the prevalence might be largely variable especially if the country is large geographically or population wise. In case of a nationally representative priori data on the prevalence is available and the screening of CH in a prospective study is infeasible, the nationally representative data may be used as the proxy. Prevalence score should be deducted as described in the methods section.
- If malaria is endemic over a large part of the country/population, the country-specific proportion of the global malaria caseload based on annual global malaria report of the WHO needs to be referred, and the corresponding score for malaria should be included in the extended reporting.
- In any study/survey that estimates anaemia, the quantitative measurement of commonly eaten ASF (i.e. cooked) in the setting is recommended. To measure the intake, any validated assessment method can be used according to suitability to the context and resources, e.g. 7-d semi quantitative FFQ, 24-h recall with multiple passes and dietary weighing method.
- Consideration of the factors for the extended reporting will vary according to the context. The intake of ASF is a ubiquitous element for Hb status; therefore, it should be considered irrespective of the settings. Similarly, congenital Hb disorders need consideration in any setting due to its presence across the countries with varied magnitude and for its profound negative influence on Hb status. Other factors for the reporting, e.g. malaria, GWI need consideration, in the cases they are recognised as significant factors for anaemia in the setting.
- For the ASF assessment, it is important to avoid isolated extreme values of consumption, as these might influence the estimates of the 97·5^th^ percentile intake. The 97·5^th^ percentile intake is the basis of the calculation of the proportion ranges of the sub-groups. The isolated extreme values might influence the positioning of the weighted-mean intake over the sub-groups, thus might pose difficulties in comparison of the ASF scores between the settings or in the same setting over the temporal time points. Imputation of the isolated extreme values with the mean value of the intake may redress this somewhat^(48)^.
- Blood sample collection for Hb measurement should be consistent, as there is a wide variation in the estimates of anaemia between capillary and venous sampling of the blood sample. The capillary sampling is usually linked with overestimation of the prevalence of anaemia^(20,49)^. Inconsistency of blood sampling over the temporal time points or between the settings would likely to make the interpretation of the extended reporting difficult.

### Strength/limitation

Strength of the study is that it is conducted analysing two nationally representative data and three population groups that are considered. The findings across the population groups are consistent. The usage of Hb values linked with the inflammation adjusted ferritin reduces the possibility of inflammation to affect the Hb which adds to the strength of the study. Studies have shown that the capillary methods of blood sampling overestimate anaemia prevalence^(20,49)^. The usage of venous blood to measure Hb is an additional strength of the study. The measured concentration of GWI is not from the direct/actual source of drinking of the survey participants (i.e. tube wells of the households), but is the average concentration of the sub-districts where they reside. This constitutes a limitation of the study.

## Conclusion

An extended reporting of anaemia beyond the prevalence estimate can inform the status of the context-specific principal influencing factors of anaemia. In Bangladesh, the measurement of the intake of ASF, GWI concentration and the magnitude of congenital Hb disorders are useful components of the reporting. The caseload of malaria in settings where malaria is endemic can be included. The reporting may suggest the key actions to control anaemia, identify the actions needing priority as well as the actions needing a cautious approach and adjustment. The reporting is potentially useful to compare different settings in terms of the key correlates of anaemia and can be used to monitor the progress of the intervention for anaemia control in a given setting.

## Supplementary material

For supplementary material accompanying this paper visit https://doi.org/10.1017/S1368980023000393. click here to view supplementary material

## References

[1] World Health Organization (2020) WHO Global Database on Anaemia. Worldwide Prevalence of Anaemia 1993–2005. Geneva: WHO/CDC.

[2] World Health Organization (2011) Haemoglobin Concentrations for the Diagnosis of Anaemia and Assessment of Severity. Vitamin and Mineral Nutrition Information System. Geneva: World Health Organization (WHO/NMH/NHD/MNM/11.1).

[3] Stoltzfus RJ (2003) Iron deficiency: global prevalence and consequences. Food Nutr Bull 24, S99–S103. 17016951 10.1177/15648265030244S206

[4] Balarajan Y , Ramakrishnan U , Özaltin E et al. (2011) Anaemia in low-income and middle-income countries. Lancet 378, 2123–2135. 21813172 10.1016/S0140-6736(10)62304-5

[5] Muriuki JM , Mentzer AJ , Mitchell R et al. (2021) Malaria is a cause of iron deficiency in African children. Nat Med 27, 653–658. 33619371 10.1038/s41591-021-01238-4 PMC7610676

[6] White NJ (2022) What causes malaria anemia? Blood 139, 2268–2269. 35420692 10.1182/blood.2021015055

[7] Merrill RD , Shamim AS , Ali H et al. (2011) Iron status of women is associated with the iron concentration of potable groundwater in rural Bangladesh. J Nutr 141, 944–949. 10.3945/jn.111.138628 21451130

[8] Rahman S , Ahmed T , Rahman AS et al. (2016) Determinants of iron status and Hb in the Bangladesh population: the role of groundwater iron. Public Health Nutr 19, 1862–1874. 26818180 10.1017/S1368980015003651 PMC10270950

[9] Rahman S , Lee P , Raqib R et al. (2019) Effect of micronutrient powder (MNP) with a low-dose of iron on haemoglobin and iron biomarkers, and its effect on morbidities in rural Bangladeshi children drinking groundwater with a high-level of iron: a randomized controlled trial. Nutrients 11, 2756. 31766250 10.3390/nu11112756 PMC6893643

[10] Karakochuk CD , Whitfield KC , Barr SI et al. (2015) Genetic hemoglobin disorders rather than iron deficiency are a major predictor of hemoglobin concentration in women of reproductive age in rural Prey Veng, Cambodia. J Nutr 145, 134–142. 25527668 10.3945/jn.114.198945

[11] Sanchaisuriya K , Fucharoen S , Ratanasiri T et al. (2006) Thalassemia and hemoglobinopathies rather than iron deficiency are major causes of pregnancy-related anemia in northeast Thailand. Blood Cells Mol Dis 37, 8–11. 16750922 10.1016/j.bcmd.2006.04.006

[12] Mistry SK , Akter F , Mukta US et al. (2015) Exploration of Multiple Micronutrient Powder (MNP) Usage among Children of 6–59 Months in Bangladesh MIYCN Home Fortification Program (MIYCN Phase II Areas). Working Paper. https://www.academia.edu/31070581/Exploration_of_Multiple_Micronutrient_Powder_MNP_usage_among_Children_of_6_59_months_in_Bangladesh_MIYCN_Home_Fortification_Programme_MIYCN_Phase_II_Areas (accessed December 2021).

[13] Mishra AK & Tiwari A (2013) Iron overload in thalassaemia major and intermedia patients. Maedica 8, 328–332. 24790662 PMC3968466

[14] (2013) National Micronutrient Survey 2011–2012; Final Report. Dhaka: Institute of Public Health Nutrition, United Nation Children’s Fund, icddr,b and Global Allaince for Improved Nutrition.

[15] (2021) National Micronutrient Survey 2020–2021; Preliminary Report. Bangladesh: National Nutrition Service, GoB and icddr,b.

[16] Bangladesh Bureau of Statistics and UNICEF Bangladesh (2019) Progotir Pathey, Bangladesh Multiple Indicator Cluster Survey 2019, Survey Findings Report. Dhaka: Bangladesh Bureau of Statistics.

[17] Shaheen N , Amin R , Islam S et al. (2021) Nutrient Density and Affordability of Habitual and Desirable Diets in Bangladesh by Life Cycle Stage, Region, and Vulnerable Groups. Bangladesh: Institute of Nutrition and Food Science and Institute of Health Economics, University of Dhaka.

[18] Hurrell R & Egli I (2010) Iron bioavailability and dietary reference values. Am J Clin Nutr 91, 1461S–1467S. 20200263 10.3945/ajcn.2010.28674F

[19] (2021) Cooley’s Anaemia Foundation 330 Seventh Avenue, #200 New York, NY 10001. www.cooleysAnaemiafoundation.org (accessed November 2021).

[20] Rahman S & Ireen S (2019) Groundwater iron has the ground: low prevalence of anaemia and iron deficiency anaemia in Bangladesh, Am J Clin Nutr 110, 519–520. 31095289 10.1093/ajcn/nqz052

[21] Choudhury N , Siddiqua TJ , Ahmed SMT et al. (2021) Iron content of drinking water is associated with anaemia status among children in high groundwater iron areas in Bangladesh. Trop Med Int Health 27(2), 149–157. doi: 10.1111/tmi.13710. 34905267

[22] Wendt AS , Waid JL & Gabrysch S (2019) Dietary factors moderate the relation between groundwater iron and anaemia in women and children in rural Bangladesh. Curr Dev Nutr 3, nzz093. 31620671 10.1093/cdn/nzz093 PMC6785699

[23] Madhu GN , Siva Saranappa SB & Manasa G (2017) Association between low hemoglobin level, vitamin D deficiency and acute lower respiratory tract infections in children aged 6 months to 5 years. Curr Pediatr Res 21, 181–185.

[24] Rahman S & Shaheen N (2021) An alternative approach to adjust the iron status for inflammation in population: an exploratory study. World Nutr 12, 51–69.

[25] (2020) Thalassaemia. https://www.britannica.com/science/haemoglobin (accessed January 2022).

[26] Adegbola AT , Havelaar AH , McKune SL et al. (2020) Animal source foods: sustainability problem or malnutrition and sustainability solution? Perspective matters. Glob Food Sec 25, 100325.

[27] British Geological Survey, Department for Public Health Engineering & Government of the People’s Republic of Bangladesh (2001) Arsenic Contamination of Groundwater in Bangladesh. BGS Technical Report WC/00/19, pp. 8–21 [ DG Kinniburgh and PL Smedley , editors]. Keyworth: BGS.

[28] Modell B & Darlison M (2008) Global epidemiology of haemoglobin disorders and derived service indicators. Bull World Health Organ 86, 480–487. 18568278 10.2471/BLT.06.036673 PMC2647473

[29] Flint J , Harding RM , Boyce AJ et al. (1998) The population genetics of the haemoglobinopathies. Baillière’s Clin Haematol 11, 1–51. 10872472 10.1016/s0950-3536(98)80069-3

[30] Merrill RD , Shamim AA , Ali H et al. (2012) High prevalence of anaemia with lack of iron deficiency among women in rural Bangladesh: a role for thalassaemia and iron in groundwater. Asia Pac J Clin Nutr 21, 416–424. 22705433

[31] National Institute of Population Research and Training & ICF (2019) Bangladesh Demographic and Health Survey 2017–2018: Key Indicators. Dhaka, and Rockville, MD: NIPORT and ICF.

[32] Namaste SML , Aaron JA , Varadhan R et al. (2017) Methodologic approach for the biomarkers reflecting inflammation and nutritional determinants of anaemia (BRINDA) project. Am J Clin Nutr 106, Suppl. 1, 333S–347S. 28615254 10.3945/ajcn.116.142273 PMC5490643

[33] Noor FA , Sultana N , Bhuyan GS et al. (2020) Nationwide carrier detection and molecular characterization of *β*-thalassaemia and haemoglobin E variants in Bangladeshi population. Orphanet J Rare Dis 15, 15. 31941534 10.1186/s13023-020-1294-z PMC6961315

[34] Rahman S , Lee P , Ireen S et al. (2021) Validation of an interviewer-administered 7-d semi-quantitative food frequency questionnaire for the dietary assessment of preschool children in rural Bangladesh. J Nutr Sci 10, e26. 33996039 10.1017/jns.2021.19 PMC8080233

[35] Shaheen N , Rahim ATMA , Mohiduzzaman M et al. (2013) Food Composition Table for Bangladesh. Dhaka: Institute of Nutrition and Food Science Centre for Advanced Research in Sciences, University of Dhaka.

[36] Richter M , Baerlocher K , Bauer JM et al. (2019) Revised reference values for the intake of protein. Ann Nutr Metab 74, 242–250. 30904906 10.1159/000499374 PMC6492513

[37] Fraenkel PG (2017) Anaemia of inflammation: a review. Med Clin North Am 101, 285–296. 28189171 10.1016/j.mcna.2016.09.005 PMC5308549

[38] Ganz T (2019) Anaemia of inflammation. N Engl J Med 381, 1148–1157. 31532961 10.1056/NEJMra1804281

[39] Khan WA , Banu B , Amin SK et al. (2005) Prevalence of beta thalassaemia trait and Hb E trait in Bangladeshi school children and health burden of thalassaemia in our population. DS HJ 21, 1–7.

[40] Ahmed F , Khan MR , Chowdhury IA et al. (2019) Effect of routine iron–folic acid supplementation among rural pregnant women living in low- and high-groundwater-iron areas in Bangladesh. Public Health Nutr 22, 2844–2855. 31274069 10.1017/S1368980019001617 PMC10260539

[41] Colah R. , Goraksakhar A & Nadkarni A (2010) Global burden, distribution and prevention of *β*-thalassaemia and haemoglobin E disorders. Expert Rev Hematol 3, 103–117. 21082937 10.1586/ehm.09.74

[42] Eads A & Alpeche J (2022) How to calculate weighted average in 3 steps. https://www.indeed.com/career-advice/career-development/how-to-calculate-weighted-average (accessed August 2022).

[43] Saito H (2014) Metabolism of iron stores. Nagoya J Med Sci 76, 235–254. 25741033 PMC4345694

[44] Atanasiu V , Manolescu B & Stoian I (2007) Hepcidin – central regulator of iron metabolism. Eur J Haematol 78, 1–10. 17042775 10.1111/j.1600-0609.2006.00772.x

[45] Jaeggi T , Kortman GA , Moretti D et al. (2015) Iron fortification adversely affects the gut microbiome, increases pathogen abundance and induces intestinal inflammation in Kenyan infants. Gut 64, 731–742. 25143342 10.1136/gutjnl-2014-307720

[46] Zimmermann MB , Chassard C , Rohner F et al. (2010) The effects of iron fortification on the gut microbiota in African children: a randomized controlled trial in Cote d’Ivoire. Am J Clin Nutr 92, 1406–1415. 20962160 10.3945/ajcn.110.004564

[47] WHO (2021) World Malaria Report 2021. Geneva: World Health Organization.

[48] Glas CAW (2010) Imputation Method; in International Encyclopedia of Education, 3rd ed. https://www.sciencedirect.com/topics/mathematics/imputation-method (accessed December 2021).

[49] Neufeld LM , Larson LM , Kurpad A et al. (2019) Haemoglobin concentration and anaemia diagnosis in venous and capillary blood: biological basis and policy implications. Ann N Y Acad Sci 1450, 172–189. 31231815 10.1111/nyas.14139 PMC7496102

