# Supplementary material for: Behind the scene of the prevalence of anaemia: an extended way of reporting
Source: Public Health Nutr. 2023 Feb 27;26(6):1115–24. doi: 10.1017/S1368980023000393 (PMC10346090; doi:10.1017/S1368980023000393)
Supplement: Supplementary file 1 [file S1368980023000393sup001.docx]

Supplementary Table 1: Correlation of haemoglobin concentration vs. animal source- iron, zinc and vitamin A

|  | Haemoglobin with ferritin unadjusted for inflammation | | |
| --- | --- | --- | --- |
|  | Haemoglobin vs. animal source micronutrients | | |
| **Population** | n | Kendall’s tau b | p-value |
| **PSC** |  |  |  |
| Animal source iron | 457 | 0.015 | 0.62 |
| Animal source vitamin A | 569 | 0.009 | 0.78 |
| Animal source zinc |  | 0.023 | 0.46 |
| **SAC** |  |  |  |
| Animal source iron | 1298 | 0.06 | 0.001 |
| Animal source vitamin A | 1323 | 0.05 | 0.004 |
| Animal source zinc | 1323 | 0.07 | 0.003 |
| **NPNLW** |  |  |  |
| Animal source iron | 1036 | 0.015 | 0.46 |
| Animal source vitamin A | 1036 | 0.008 | 0.69 |
| Animal source zinc | 1036 | 0.025 | 0.23 |

Legend: PSC: Preschool Children (2-5 years-old), SAC: School Age Children (6-14 years), NPNLW: Non-pregnant Non-lactating women (15-49 years)

Supplementary Table 2: **Intake of ASF at the 97.5^th^ and 100^th^ percentiles in the Bangladeshi populations**

| ASF/day (percentile) | n | Intake of ASF (g/d) | 95% CI |
| --- | --- | --- | --- |
| **PSC** |  |  |  |
| 97.5^th^ | 1037 | **449.34** | 388.4, 516.3 |
| 100^th^ | 1037 | 1035.9 | 1035.9, 1035.9 |
| **SAC** |  |  |  |
| 97.5^th^ | 1430 | **426.43** | 395.54, 446.26 |
| 100^th^ | 1430 | 3881.7 | 3881.7, 3881.7 |
| **NPNLW** |  |  |  |
| 97.5^th^ | 1418 | **395.07** | 374.64, 425.42 |
| 100^th^ | 1418 | 613.48 | 613.48, 613.48 |

Legend: PSC: Preschool Children (2-5 years-old), SAC: School Age Children (6-14 years), NPNLW: Non-pregnant Non-lactating women (15-49 years)

**B.NPNLW**

**A.PSC**

**Ferritin (ng/ml**

**Ferritin (ng/ml**

Supplementary Figure 1: The mspline graphs showing the relation of the groundwater iron concentrations (mg/L) and the inflammation-adjusted serum ferritin (ng/ml).

Legend: PSC: Preschool Children (2-5 years-old), NPNLW: Non-pregnant Non-lactating women (15-49 years)

The mspline graphs of groundwater iron concentration vs. serum ferrritin concentrations show that at around the 8-11 mg/L of the groundwater iron concentration there is a steady [PSC] and a profound [NPNLW] trend of rise of the inflammation-adjusted serum ferritin.

Legend: Preschool children (PSC); Non-pregnant Non-lactating women (NPNLW); GW Fe Conc. : Groundwater iron concentration (mg/L)

**GW Fe conc.**

**GW Fe conc.**
